# Sequence edition of single domains modulates the final immune and antimicrobial potential of a new generation of multidomain recombinant proteins

**DOI:** 10.1038/s41598-021-03220-z

**Published:** 2021-12-10

**Authors:** Ramon Roca-Pinilla, Ravi Holani, Adrià López-Cano, Cristina Saubi, Ricardo Baltà-Foix, Eduardo R. Cobo, Elena Garcia-Fruitós, Anna Arís

**Affiliations:** 1 Department of Ruminant Production, Institut de Recerca i Tecnologia Agroalimentàries (IRTA), 08140 Caldes de Montbui, Spain; 2 Department of Production Animal Health, Faculty of Veterinary Medicine, University of Calgary, Calgary, AB Canada

**Keywords:** Biotechnology, Microbiology

## Abstract

Combining several innate immune peptides into a single recombinant antimicrobial and immunomodulatory polypeptide has been recently demonstrated. However, the versatility of the multidomain design, the role that each domain plays and how the sequence edition of the different domains affects their final protein activity is unknown. Parental multidomain antimicrobial and immunomodulatory protein JAMF1 and several protein variants (JAMF1.2, JAMF2 and AM2) have been designed and recombinantly produced to explore how the tuning of domain sequences affects their immunomodulatory potential in epithelial cells and their antimicrobial capacity against Gram-positive and Gram-negative bacteria. The replacement of the sequence of defensin HD5 and phospholipase sPLA_2_ by shorter active fragments of both peptides improves the final immunomodulatory (IL-8 secretion) and antimicrobial function of the multidomain protein against antimicrobial-resistant *Klebsiella pneumoniae and Enterococcus *spp. Further, the presence of Jun and Fos leucine zippers in multidomain proteins is crucial in preventing toxic effects on producer cells. The generation of antimicrobial proteins based on multidomain polypeptides allows specific immunomodulatory and antimicrobial functions, which can be easily edited by modifying of each domain sequence.

## Introduction

The continuous rise in drug-resistant microbes is already challenging the treatment of infections and has put health authorities on alert. Antimicrobial resistances (AMRs) are one of the health threats included within the One Health approach, which aims to find solutions to the appearance and prevalence of AMRs by cooperation across all sectors, including human, animal and environmental health^1^. In the search for new antimicrobials, several strategies have been studied including developing anti-infectious molecules based on natural compounds, such as essential oils or flavonoids, probiotics and prebiotics, and bacteriophages, among others^2^. However, most of them present important drawbacks such as low antimicrobial efficiencies, challenging and time-consuming procedures for their isolation and/or narrowed antimicrobial spectra, or a combination of several of these handicaps. Taking a new perspective in the development of antimicrobial molecules, the use of active peptides or proteins naturally present in the innate and adaptive immunity offers a powerful strategy to develop new antimicrobial molecules^3,4^. Among them, there are larger antimicrobial proteins formed by more than 100 amino acids which are often lytic enzymes, nutrient-binding proteins, or proteins containing sites that target specific microbial macromolecules^5^. The smaller antimicrobial peptides, also known as host defense peptides (HDPs), are cationic, amphiphilic and short peptides synthesized by nearly all multicellular organisms with activity against bacteria, viruses and fungi^6^. So far, HDPs have been intensively explored, proving that they offer a great potential to treat a wide range of microorganisms, including multidrug-resistant strains, and some of them also present immunomodulatory effects^7^. Most of this research has been done using chemically synthesized peptides. The production of such peptides, however, is expensive and intrinsically ineffective at a large scale, thus limiting the clinical application of these molecules^8^. The recombinant production of antimicrobial peptides and proteins from the immune system, whereas promising, is still challenging. The small size of HDPs impairs their stability in front of proteases, and its antimicrobial nature triggers toxic effects on the recombinant producer bacteria, usually blocking an efficient recombinant production. Different groups have addressed the fusion of a carrier protein to the antimicrobial peptide of interest to minimize the toxicity and increase their stability^9–12^. This option, however, comprises a tedious and cost-ineffective work of carrier protein removal. To overcome these limitations, our group has recently published a new strategy based on the synthesis of several antimicrobial proteins and peptides combined in a single polypeptide that codifies a multidomain antimicrobial protein named JAMF1^13^. This approach allows to recombinantly produce antimicrobial peptides of interest at good yields, with no toxicity for the producer bacteria, and without the need of adding non-functional carrier proteins^13^. The multidomain protein JAMF1 combined the HDP human α-defensin-5 (HD5), a bacterial binding domain (gelsolin), and an enzymatic antimicrobial peptide (sPLA_2_), flanked by two aggregation-seeding domains (a fragment of c-Jun and c-Fos leucine zippers at N- and C-terminal, respectively) in a single polypeptide. We demonstrated that the resulting antimicrobial multidomain protein showed activity against both Gram-positive and Gram-negative drug-resistant bacteria^13^. However we did not study if the domains were switchable or editable in a manner that the final function of the polypeptide could be modulated as needed. If this could be proven, a battery of antimicrobial proteins could be easily generated to be used in several applications such as different pathologies and clinical profiles requiring immunomodulation and antimicrobial effects simultaneously, among others. In this context, the main aim of the present work is to explore whether the sequence edition of JAMF1 domains can be performed individually to finally improve the immunostimulant and/or antimicrobial function of the whole multidomain protein.

## Results

### Constructs design and protein production

**Figure 1:**
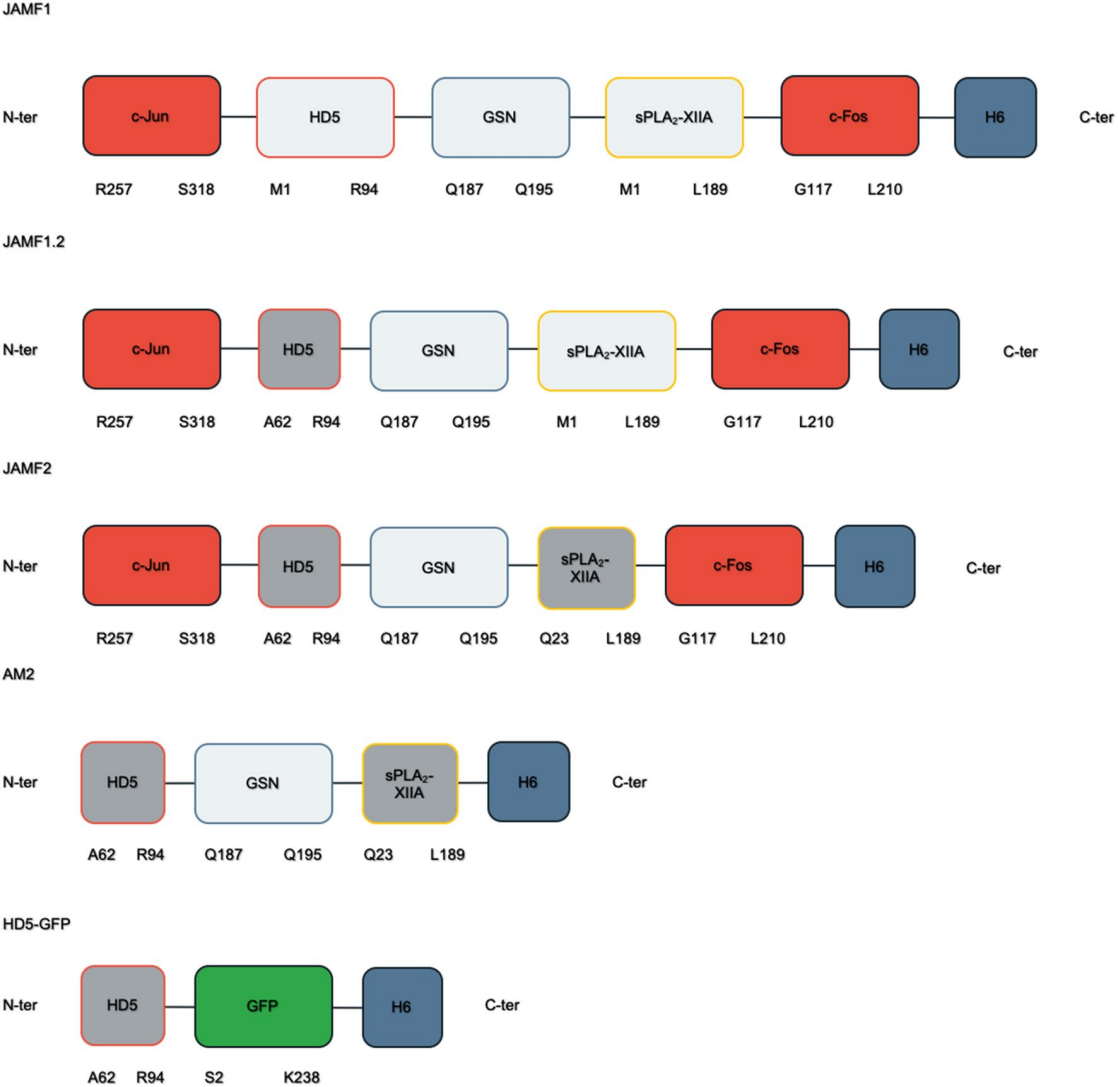
Schematic representation of the different antimicrobial multidomain proteins. The construct JAMF1 contains the whole amino acid sequences of the HD5 and sPLA_2_-XIIA domains, whereas JAMF1.2 contains the whole amino acid sequence of sPLA_2_-XIIA but a fragment (A62-R94) of the HD5. Instead, JAMF2 has the A62-R94 fragment of HD5 and the mature form of sPLA_2_-XIIA domains, and the AM2 construct is identical to JAMF2 without the leucine zipper domains (c-Jun and c-Fos). Finally, the HD5 construct contains the A62-R94 fragment of HD5 coupled to a GFP as a carrier protein to allow its recombinant production. All constructs have six histidine residues at the C-terminal for protein purification.

**Figure 2:**
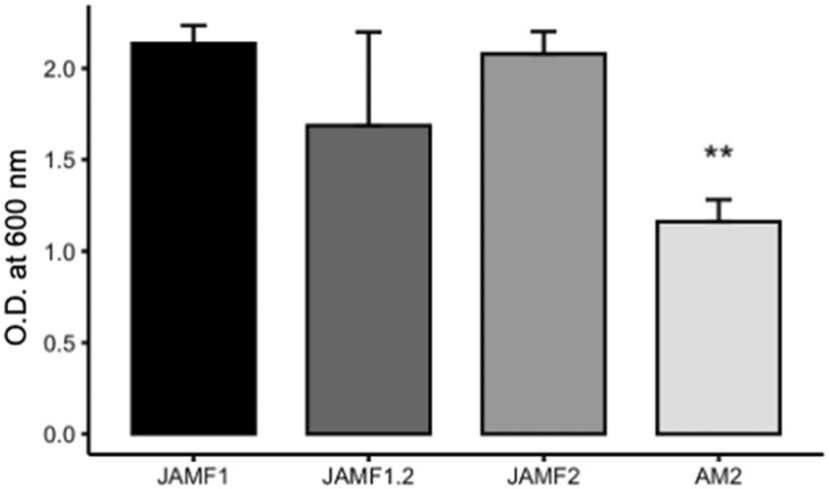
Biomass measure (OD_600_) of *E. coli* BL21 at 3 h of production of the proteins JAMF1, JAMF1.2, JAMF2, which contain the aggregation tags Jun and Fos, and the protein AM2, without the aggregation tags. ** Shows a statistically significant difference (*p* < 0.01).

Several JAMF1 protein derivatives (Fig. 1) were designed and produced to evaluate how sequence changes in single domains could influence the antimicrobial and immunomodulatory activity of the recombinant multidomain protein and to explore if changes in different domains could have a final accumulative effect. While the parental JAMF1 construct had the complete sequences of HD5 and sPLA_2_ containing the signal peptides, JAMF1.2, JAMF2, AM2 and HD5-GFP constructs contained a shorter fragment of HD5 sequence corresponding to HD5 63-94. JAMF2 and AM2 also presented the mature form of sPLA_2_. All multidomain proteins contained Jun and Fos tags, except AM2 protein. All proteins were produced in *Escherichia coli* mainly as inclusion bodies (IBs), which after mild solubilization and purification lead to soluble protein in a yield that ranged from 0.3 to 2.7 mg/L culture. Instead, AM2 showed toxic effects for the producer *E. coli* strain (Fig. 2), achieving just low protein yields (0.001 mg/L).

### Immunomodulatory activity in colonic epithelial cells

**Figure 3:**
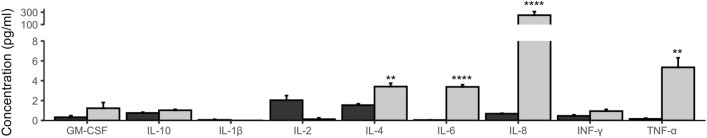
Secretion of different cytokines by CaCo2 cells non-exposed (black bars, control) and exposed to JAMF2 1 μM (grey bars). **p < 0.01; ****p < 0.0001 treated versus control cells.

**Figure 4:**
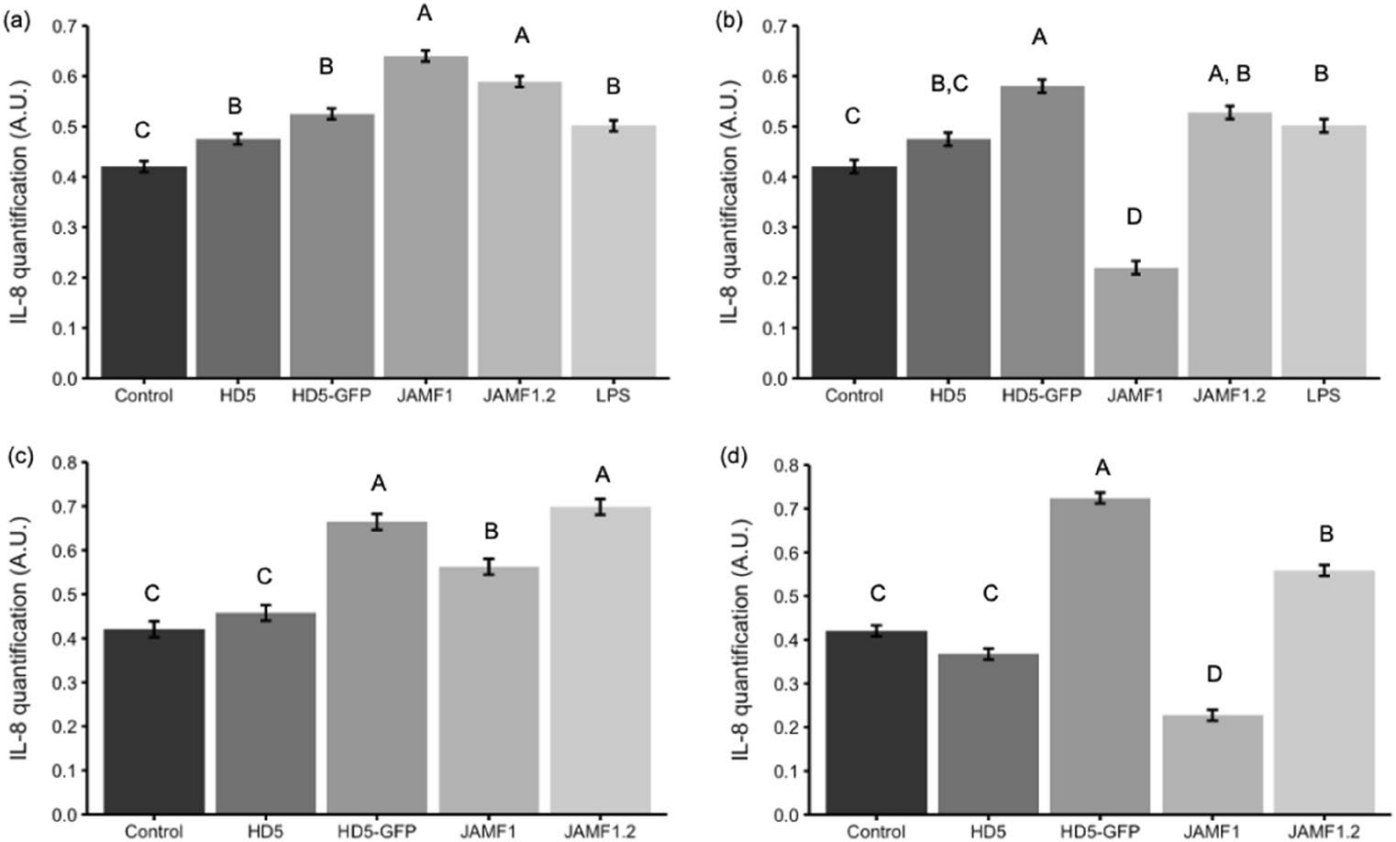
IL-8 secretion (absorbance units (A.U.)) by colonic epithelial HT-29 cells treated during 24 h with 0.1 (**a**,**c**) or 1 μM (b, d) of the different proteins in the presence (**a**,**b**) or absence (**c**,**d**) of LPS (1 μg/mL). All treatments have been compared by ANOVA analysis. Different capital letters indicate statistically significant differences (*p* ≤ 0.0001).

To have a preliminary idea of the extent of the immunomodulatory effects of multidomain proteins an experiment using JAMF2 was performed by Luminex analyses in colon cell cultures. Significant increases in the secretion of IL-4, IL-6, IL-8 and TNF-α cytokines were found, being the IL-8 increase the greatest one (Fig. 3). Hence, IL-8 was used as a marker to compare the activity of multidomain proteins after domain sequence edition. In these comparative experiments, HT29 cells were challenged (or not) with LPS while incubated with the proteins. A different performance of the proteins was observed depending on the presence of LPS stimuli and protein concentrations (Fig. 4). Under LPS challenge and at low protein concentration (Fig. 4a), both multidomain proteins JAMF1 (with the complete HD5 sequence) and JAMF1.2 (containing the A62-R94 fragment of HD5) induced greater IL-8 secretion than proteins with a single HDP (HD5-GFP and synthetic HD5), which did not show any increase on IL-8 secretion compared to cells only incubated with LPS. Without LPS stimulus and low protein concentration (Fig. 4c), the recombinant proteins containing the A62-R94 fragment of HD5, either the HD5-GFP or multidomain JAMF1.2 proteins, induced the greatest secretion of IL-8. Synthetic HD5 peptide did not increase IL-8 secretion compared to the control (Fig. 4c). At a higher concentration (Fig. 4b,d), all the proteins performed in a similar way as observed at lower protein concentration (Fig. 4a,c), except for JAMF1 that inhibited IL-8 secretion independently of the presence of LPS challenge (Fig. 4b,d).

### Antimicrobial and enzymatic activity

**Figure 5:**
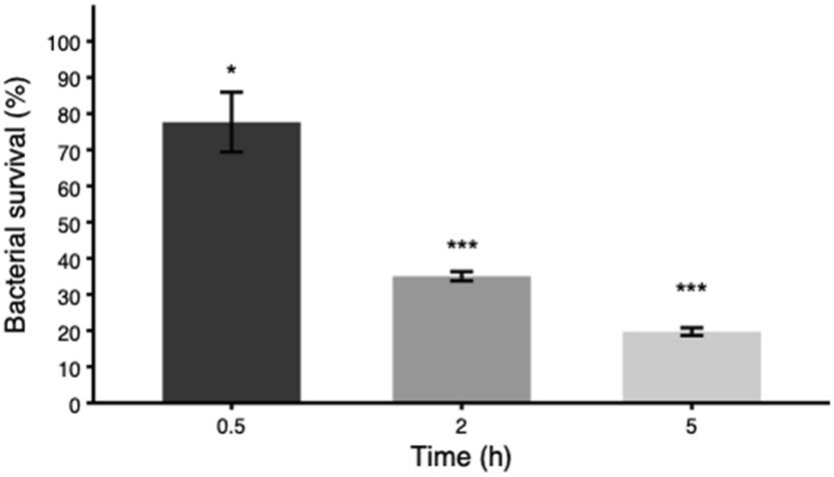
Survival of carbapenem-resistant *K. pneumoniae* over time after being exposed to the antimicrobial multidomain protein JAMF2 at a concentration of 5 μM. *, *** Show a statistically significant significant differences (*p* < 0.05, and *p* < 0.001, respectively).

**Figure 6:**
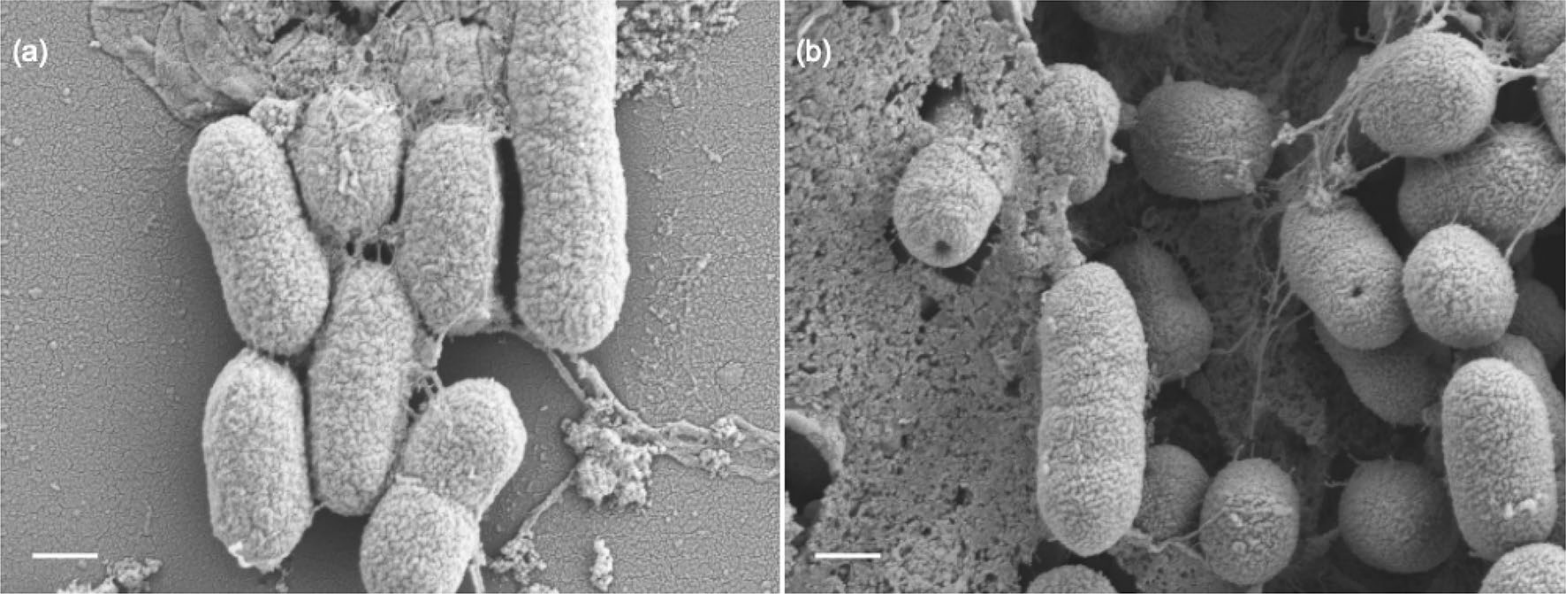
Representative high-resolution FESEM images of *K.pneumoniae*. Untreated control (**a**) and bacteria exposed to 5 μM of JAMF2 for 5 min (**b**). Bar size: 500 nm.

**Figure 7:**
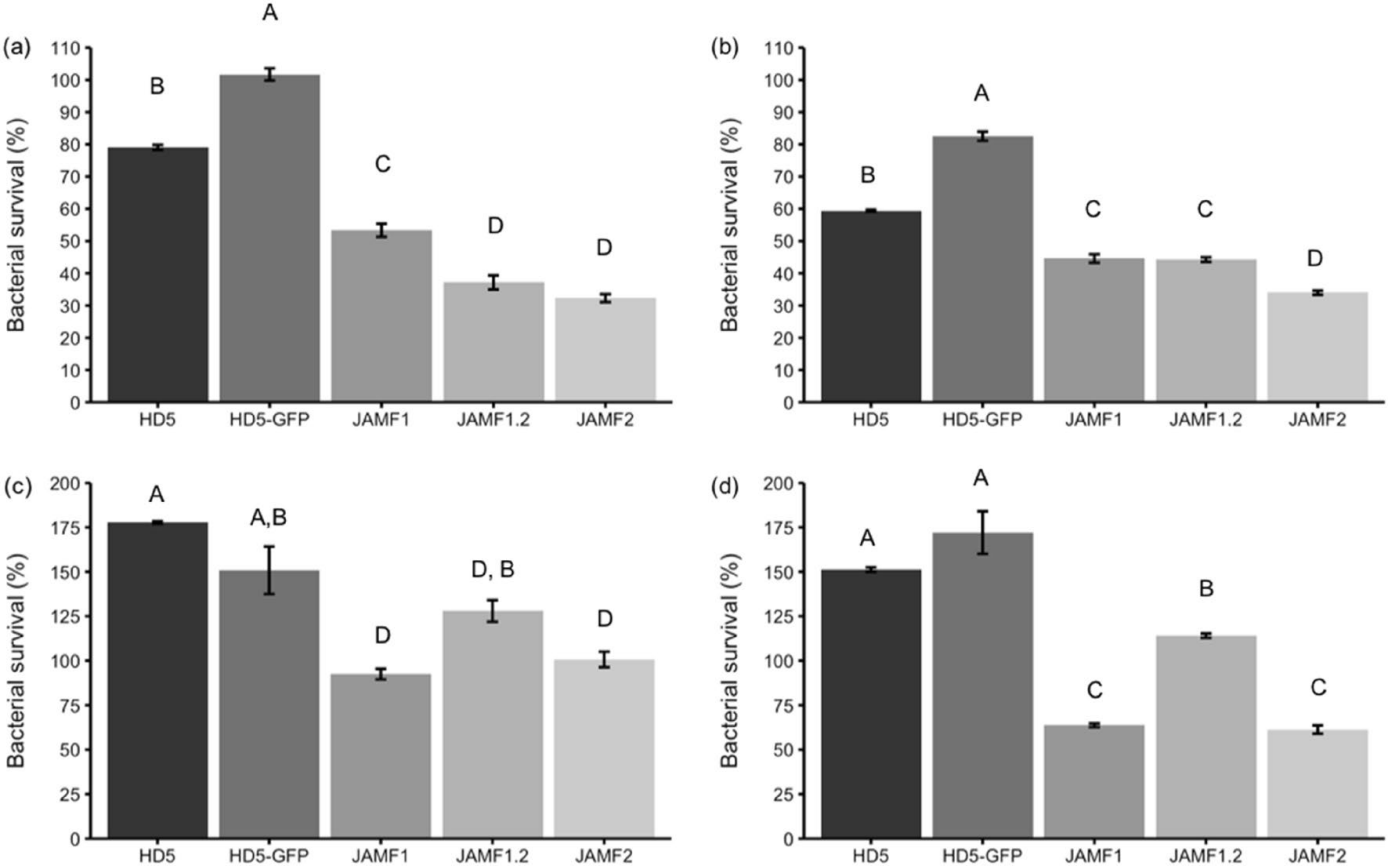
Antimicrobial activity of the different antimicrobial constructs at 1 (**a**,**c**) and 3 μM (**b**,**d**) against carbapenem-resistant *Klebsiella pneumoniae* (**a**,**b**) and *Enterococcus *spp. CTX-M-14 (**c**,**d**). All treatments have been compared by ANOVA analysis. Different capital letters indicate statistically significant differences (*p* ≤ 0.0001).

**Figure 8:**
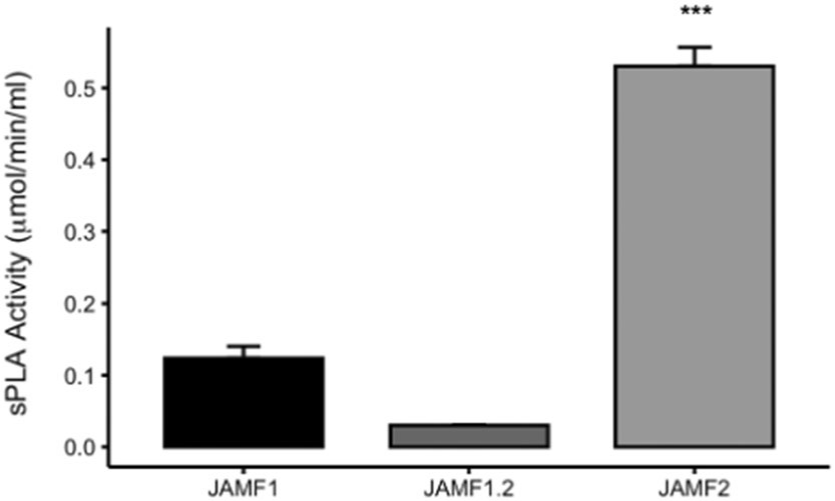
Enzymatic activity of the secreted phospholipase (sPLA_2_) domain for each antimicrobial construct. Asterisks show a statistically significant difference (*p* < 0.001).

The optimum time to evaluate the antimicrobial activity of these multidomain proteins was assessed using *Klebsiella pneumoniae* (KPC) as an indicator (Fig. 5). A time of 5 h was the best incubation time tested to observe the maximum bactericidal activity. However the antimicrobial activity started in a few minutes since SEM micrographs of KPC showed clear pores in the bacteria cell wall after 5 min of incubation with JAMF2 protein (Fig. 6). After this preliminar experiment, 5-h incubation was used to compare the antimicrobial performance of recombinant constructs and synthetic HD5 peptides. Proteins and peptides were evaluated against Gram-negative (KPC) and Gram-positive bacteria (*Enterococcus *spp. CTX-M-14 (EC)) at two different concentrations (1 and 3 μM) (Fig. 7). The antimicrobial activity was greater against Gram-negative KPC reducing the bacterial survival up to 70% (Fig. 7a,b). The most active antimicrobial protein was JAMF2 (at 1 or 3 μM) followed by JAMF1.2 and JAMF1. By contrast, the EC was slightly inhibited (40%) only at 3 μM by JAMF1 and JAMF2 (Fig. 7c,d). HD5 synthetic peptide was more bactericidal against KPC than the recombinant HD5 peptide fused to GFP. The analysis of the enzymatic activity of the sPLA_2_ domain showed greater values for JAMF2 (containing sPLA_2_ mature sequence) than for JAMF1.2 and JAMF1 (Fig. 8).

## Discussion

We have recently described a novel and versatile strategy to create easily editable multidomain proteins^13^. The parental construct, named JAMF1, linked up the HDP human alfa defensin 5 (HD5), a bacterial binding domain (gelsolin) and an enzymatic antimicrobial peptide (sPLA_2_), flanked by two aggregation-seeding domains (c-Jun and c-Fos leucine zippers fragments at N- and C-terminal, respectively)^13^. HD5 is the major antimicrobial defensin peptide of ileal Paneth cells whose mechanism of action is based on pore-forming activity in the bacterial cell wall. Moreover, HD5 has been described as an antimicrobial peptide with immunomodulatory properties. Besides, sPLA_2_ is an enzyme involved in the host’s defense via phospholipid degradation of bacterial membranes^14^. Thus, both HD5 and sPLA_2_ are peptides able to disrupt bacterial cells, but HD5, like other HDPs, also has an important role contributing to the regulation of host immunity. HD5 is responsible for up-regulating the expression of genes such as IL-8, IL-2 and IFN-γ involved in cell survival and inflammation in an NF-kB-dependent fashion in epithelial cells^15^. Herein we have described that JAMF-derived constructs can activate the release of IL-4, IL-6, IL-8 and TNF-α cytokines on colonic cells but not IL-2 or IFN-γ (Fig. 3) Likewise, gelsolin domain is involved in the host immune recognition of lipoteichoic acid (LTA) and LPS of Gram-positive and Gram-negative bacteria, respectively, and its presence in the multidomain polypeptides aims to improve the targeting to pathogens^16^. Incorporating of two seeding domains such as Jun and Fos leucine zippers fosters protein aggregation inside the recombinant producer bacteria, but does not exert any activity against pathogens.

In our previous study^13^ we demonstrated that combining several peptides from the innate immunity in a single recombinant polypeptide is possible. In this study, we showed that the multidomain design is versatile and the activity of the construct could be tuned by slight changes in the sequences of each domain which could have a cumulative effect on the final multidomain protein. Several JAMF1 protein variants based on the tuning of antimicrobial sequences of HD5 and sPLA_2_ domains have been designed and produced herein, leading to JAMF1.2, JAMF2 and AM2 proteins (Fig. 1). The rest of the domains, which are not antimicrobial or immunomodulatory per se, have been maintained intact in all the constructs, except for AM2, where the Jun and Fos domains were removed. As expected, AM2 was toxic to the producer cell, reducing the final biomass of the culture (Fig. 2). It seems that the seeding domains lead to the rapid formation of protein aggregates known as bacterial IBs. IBs can be directly used as an antimicrobial material^13^, but they can also be used, as is the case of this study, as a source of soluble antimicrobial proteins using a mild non-denaturing solubilization protocol previously established in our group^17^.

To evaluate the immunomodulatory effect, we compared the ability of the proteins to induce chemokine IL-8 secretion on intestinal epithelial cells (Fig. 4). The IL-8 chemokine (CXCL-1 in mice) is key when secreted by colonic epithelia in the recruitment of neutrophils into the colon during infectious colitis^18^ and it was the cytokine most secreted in our preliminary studies (Fig. 3). The results demonstrated that the whole sequence of HD5, present in JAMF1, behaved differently from the peptide fragment present in JAMF1.2. Specifically, the complete HD5 sequence led to bimodal behavior depending on the concentration used (Fig. 4). At low concentration (0.1 μM), JAMF1 increased the secretion of the IL-8 cytokine, but at a higher concentration (1 μM) it reduced the secretion of IL-8. In contrast, recombinant proteins containing a fragment of HD5 presented the same immunostimulatory effect at both concentrations (0.1 μM and 1 μM), even when accompanied with other concomitant host defense domains, as it occurred in JAMF1.2, or just fused to GFP carrier protein (HD5-GFP). Note that this experiment also pointed out the better function of HD5 when expressed recombinantly compared to the synthetic form of HD5 (Fig. 3). It is likely that when HD5 is recombinantly expressed, its folding is probably closer to its native structure, which has a clear effect on the final immunomodulatory activity of this peptide. Moreover, as expected, the capacity of the multidomain proteins to immunomodulate intestinal epithelial relied on the HD5 domain, as the inclusion of additional domains (JAMF1 and JAM1.2 compared to HD5-GFP) did not influence on its activity (Fig. 4).

We also showed that the direct antimicrobial activity of these proteins is a result of the combination of several domains. The performance of HD5-GFP was lower than that of multidomain proteins (JAMF1 and JAMF1.2, and JAMF2), which substantially inhibited bacterial pathogens (Fig. 7). Interestingly, the antimicrobial activity of synthetic HD5 peptide (Fig. 7) was not impaired as it occurred with the immunomodulatory activity (Fig. 4), indicating that the folding is not determinant for the pore-forming activity. The fact that multidomain proteins presented greater antimicrobial activity than the HD5 peptide and HD5-GFP is likely due to the presence of sPLA_2_. Indeed, among multidomain proteins containing either the premature (JAMF1 and JAMF1.2) or mature forms (JAMF2) of sPLA_2_, JAMF2 showed the highest enzymatic activity (Fig. 8). The proteins JAMF1.2 and JAMF2 sequences are equal except for the sPLA_2_ domain, that in one case is the non-mature form (JAMF1.2) while the other is the mature form (JAMF2). According to that, JAMF2 presented greater enzymatic activity (Fig. 8) and better ability to kill bacteria compared to JAMF1.2 and JAMF1 (Fig. 7). A clear-cut visual result of this activity is shown in Fig. 6 where evident pores in KPC cell wall are observed after being treated with JAMF2 only for 5 min.

This study demonstrated the cumulative effect of domain editing on the final function of antimicrobial and immunomodulatory multidomain proteins. This agrees with previous studies using synthetic peptides^19–21^ or fusion proteins^22^ where the combination of targeting domains with antimicrobial proteins allowed to develop therapies against specific pathogens. Herein, we demonstrate that not only the accumulation of new functions is possible by the addition of new domains but also by editing a domain sequence within a multidomain protein.

## Conclusions

This study demonstrates the versatility of multidomain antimicrobial and immunomodulatory proteins and the effect of sequence tuning on the final function. The combination of a specific fragment of HD5 and the mature form of sPLA_2_ improves both the immunomodulatory and antimicrobial function. We also proved that the use of Jun and Fos sequences flanking the multidomain protein allows its recombinant production, avoiding toxic effects to the producer cells.

## Methods

### Bacteria strains and growth mediums

*E. coli* BL21 (DE3) was used for recombinant protein expression. Strains used for antimicrobial activity assays were carbapenem-resistant *K. pneumoniae* (KPC) and extended-spectrum beta-lactamase producing *Enterococcus *spp. CTX-M-14 (EC) (kindly provided by Dr. Lourdes Migura-Garcia, IRTA*). E. coli* strains were grown in Luria–Bertani (LB) medium and KPC and EC were grown in Brain–Heart Infusion (BHI) broth (Scharlau).

### Genetic construct design

From N-terminal to C-terminal, the gene for the JAMF1 construct consisted of the sequences encoding Jun257-318 (Uniprot entry P05412), human α-defensin-5 (HD5) (Uniprot entry Q01523), gelsolin188-196 (Uniprot entry P06396), group-XIIA secretory phospholipase A2 (sPLA_2_) (Uniprot entry Q9BZM1) and Fos118-210 (Uniprot entry P01100)^13^. The gene encoding for the JAMF1.2 construct is identical to JAMF1 except for the human α -defensin-5, where we used HD563-94 instead. The sequence encoding for the JAMF2 construct is identical to JAMF1.2 but the sPLA_2_ domain was changed to sPLA_2_ 23-189. A linker sequence (SGGGSGGS) was used between each of the domains. For HD5-GFP, HD5 63-94 was fused to GFP^23^ using a linker sequence (GGSSRSS). In all constructs an H6-Tag was placed at the C-terminal for protein purification. The fusion constructs were codon-optimized by GeneArt (Lifetechnologies) and cloned into pET22b (Amp^R^) vector (Novagene).

### Antimicrobial protein production

All constructs were produced recombinantly in *E. coli* BL21 (DE3) using the expression vector pET22b. Cultures (1–2 L) were grown at 37 °C and 250 rpm in LB broth with ampicillin at 100 μg/ml. Protein expression was induced by 1 mM isopropyl-β-d-thiogalactoside (IPTG) at an OD_600_ = 0.4–0.6. Cultures were grown 3 h post-induction. The whole volume of each protein production was centrifuged at 6000× g and the pellet was resuspended in 120 ml of PBS 1 × with protease inhibitors (cOmplete EDTA-free, Roche). Samples of 30 ml were sonicated for 5 min at 10% amplitude (0.5 s ON/OFF cycles) for 4 rounds, resting for 5 min in ice between rounds.

### Protein solubilization and purification

Only HD5-GFP was obtained directly from the soluble fraction. After sonication, HD5-GFP was centrifuged 45 min at 15,000× g at 4 °C and the supernatant was recovered for Immobilized Metal Affinity Chromatography (IMAC) purification, as explained below. For all other constructs, protein pellets were recovered after sonication and centrifugation (45 min at 15,000× g at 4 °C) and washed twice with distilled water and then weighted. After that, pellets were solubilized under non-denaturing conditions for 40 h at room temperature (RT) under agitation with 40 ml/g of pellet of solubilization buffer (0.2% w/v N-lauroylsarcosine, 40 mM Tris and protease inhibitors), as previously described^24^. After the incubation, the supernatant was recovered through centrifugation at 15,000× g for 45 min at 4 °C for purification. Imidazole (20 mM) and NaCl (500 mM) were added to equilibrate the solubilized samples with the binding buffer composition to prepare the sample for IMAC purification. IMAC purification was performed in an ÄKTA start protein purification system (GE Healthcare) using 1 ml HisTrap HP columns (GE Healthcare). Both the binding (20 mM Tris pH = 8, 500 mM NaCl, 20 mM imidazole) and the elution buffer (20 mM Tris pH = 8, 500 mM NaCl, 500 mM imidazole) contained 0.2% N-lauroyl sarcosine. The buffer of the selected fraction was changed to 10 mM KPi (K/PO_4_ buffer, pH 7.4) with a HiTrap desalting column (GE Healthcare). Protein integrity and quantity were analyzed by SDS electrophoresis (TGX™ FastCast™, Bio-Rad), followed by western blotting with a monoclonal anti-His antibody (1:1,000, His-probe, Santa Cruz). Quantification was performed by interpolation to a standard curve of soluble T22-GFP^25^.

### Immunomodulatory activity in colonic epithelial cells

Human adenocarcinoma colonic epithelial cells (HT29 or CaCo-2) were maintained in Dulbecco’s Modified Eagle’s media (DMEM; Gibson, Life Technologies) containing 4.5 g/L glucose with 10% (v/v) fetal bovine serum (FBS; Benchmark Gemini Bio-Products), 1 mM sodium pyruvate (Gibco, Life Technologies), and 1% penicillin (100 U/ml)/streptomycin (100 μg/ml; HyClone Thermo, Fischer Scientific) in a humidified environment with 5% CO_2_ and at 37 °C. Cells were seeded in 24-well plates (Greiner, Bio-One, Monroe), cultured to 80–90% confluency and stimulated for up to 24 h in culture medium without FBS or antibiotics. CaCo-2 cultures were stimulated with recombinant JAMF2 at 1 μM and using KPi buffer as a negative control. Supernatants were collected after 24 h and simultaneous detection of multiple cytokines was performed using MILLIPLEX MAP KIT Human High Sensitivity T Cell Magnetic Bead Panel by Luminex technology. Similarly, HT29 cells were stimulated (or not) with recombinant HD5-GFP, JAMF1 or JAMF1.2 or HD5 (PeptaNova, commercial synthetic peptide, positive control) at 0.1 and 1 μM in presence or absence of LPS, 1 μg/ml for up to 24 h in culture medium without FBS or antibiotics. The dose of LPS was determined in a preliminary study using different concentrations (0.1–2 μg/ml), and in agreement with the literature^26^. Supernatants were collected from cells and levels of IL-8 determined using a DuoSet ELISA (DY208, R&D Systems).

### Antibacterial activity

Bacterial cell viability was determined with a BacTiter-Glo™ Microbial Cell Viability assay (Promega). Briefly, bacterial cells were grown O/N at 37 °C and 250 rpm and then diluted 1:100 in 10 mM KPi buffer. After that, 150 μL from the KPi diluted cells were centrifuged in 1 ml tubes at 6200× g at 4 °C for 15 min. The supernatant was removed, and the pelleted cells were resuspended with 150 μL of either KPi buffer (negative control) or 150 μL of 1 and 3 μM of the synthetic HD5 peptide or the protein constructs (HD5-GFP, JAMF1, JAMF1.2 or JAMF2). After 0.5 h, 2 h or 5 h incubation at 37 °C in a 96-well plate, 100 μL were taken and mixed with 100 μL of the BacTiter-Glo™ reagent for 5 min. Luminescence was measured in a microplate luminometer (LUMIstar®, BMG LABTECH). The measured luminescence values (arbitrary units) were normalized to the control (KPi buffer treatment; equivalent to 100% bacterial survival). HD5 (PeptaNova) served as a control.

### Scanning electron microscope (SEM)

To evaluate the mode of action of JAMF multidomain construct a SEM technique was selected. Shortly, an O/N culture of KPC was diluted 100-folds in a sterile KPi 10 mM buffer. A total of 400 μL were centrifuged (6,200× g, 15 min, 4 °C). The supernatant was removed and the bacterial pellet was resuspended in 400 μL of each treatment (KPi as a negative control and JAMF2 at 5 µM). Samples were deposited on circular coverslips and incubated in sterile 24-well plates for 5 min at 37 °C without agitation. Next, the supernatant was carefully removed. Coverslips were fixed with 2.5% glutaraldehyde in PB 0.1 M for 2 h at 4 °C, washed with PB 0.1 M, post-fixed with osmium tetroxide-potassium ferrocyanide for 2 h, washed with miliQ water, dehydrated in graded ethanol series (50, 70, 90, 96, 100%) and desiccated with HMDS. Samples were micrographed by field emission scanning electron microscopy (FESEM) Zeiss Merlin (Zeiss) running at 1 kV.

### Enzymatic assay

A fluorometric assay kit was used to measure sPLA_2_ activity (Cayman), using a sPLA_2_ substrate consisting of 1,2-dithio analog of diheptanoyl phosphatidylcholine. Upon enzymatic hydrolysis of the thio ester bond at the sn-2 position, free thiols were detected using 5,5’-dithio-bis-(2-nitrobenzonic acid) (DTNB) at 412 nm. Absorbance measurements were taken each minute during 15 min. The reaction rate (µmol/min/ml) was determined for constructs JAMF1, JAMF1.2 and JAMF2 from the absorbance change per minute of the linear portion of the curve, using the DTNB extinction coefficient. At least 8 time points were used for the calculations.

### Statistical analysis

Results are expressed as means with error bars representing standard errors of the mean (SEM). Data were obtained in triplicates and normality was assessed using a Shapiro–Wilk test. All comparisons were performed using either a two-sided unpaired Student’s t-test (asterisks) or one-way analysis of variance (ANOVA) with a post hoc Tukey HSD test for multiple group comparisons (letters). A *P* value < 0.05 was considered statistically significant. Analysis was conducted in R (R Core Team, 2019) with RStudio software (RStudio, Inc.), and figures were produced using the package ggplot2 (Wickham, 2016)^27–29^.
